# Type 2 diabetes mellitus correlates with systolic function during myocardial stress perfusion scanning with Nitrogen-13 ammonia PET

**DOI:** 10.1007/s12350-016-0482-7

**Published:** 2016-04-15

**Authors:** Luis Eduardo Juárez-Orozco, Friso M. van der Zant, Riemer H. J. A. Slart, Sergiy V. Lazarenko, Erick Alexanderson, Rene A. Tio, Remco J. J. Knol

**Affiliations:** 1 Department of Nuclear Medicine and Molecular Imaging, University Medical Center Groningen, Hanzeplein 1, Internal Post: EB50, 9700RB Groningen, The Netherlands; 2 Department of Nuclear Medicine, Medical Center Alkmaar, Alkmaar, The Netherlands; 3 PET/CT Cyclotron Unit, Faculty of Medicine, National Autonomous University of Mexico, Mexico City, Mexico; 4 Department of Cardiology, University Medical Center Groningen, Groningen, The Netherlands; 5 Biomedical Photonic Imaging Group, University of Twente, Enschede, The Netherlands

**Keywords:** PET, myocardial perfusion, diabetes mellitus, MPR, LVEF

## Abstract

**Background:**

The influence of type 2 diabetes mellitus (DM2) on systolic function is partially determined by the coronary vasodilator function, nevertheless, an independent effect is suspected. We evaluated the relationship between DM2 and systolic function considering PET quantitative myocardial perfusion.

**Methods:**

We analyzed 585 patients without a previous myocardial infarction referred to a rest and adenosine stress Nitrogen-13 ammonia PET. A bootstrapped multiple linear regression analysis was performed using DM2, stress myocardial blood flow (sMBF), myocardial perfusion reserve (MPR), and clinical risk factors as predictors and LVEF as the outcome variable; an interaction term was additionally investigated.

**Results:**

Two hundred and ninety male and 295 female patients (mean age 65.3 ± 9.9 and 67.4 ± 10 years, respectively) were included. 57.1% presented hypertension, 16% smoking, 37.6% hypercholesterolemia, 33.8% family history for CAD, and 15.2% DM2. The mean MPR was 2.13 ± 0.48 and 2.21 ± 0.60, mean sMBF was 2.01 ± 0.51 and 2.15 ± 0.54, and mean LVEF was 63% ± 10.4 and 67% ± 10.1 for diabetics and non-diabetics, respectively. A significant relation was detected for sMBF (*B* = 5.830 95% CI [3.505, 9.549], *P* = .001) and DM2 (*B* = −2.599 95% CI [−5.125, −0.119], *P* = .03) with LVEF. The interaction (DM2 × sMBF) yielded no significance (*P* = .512).

**Conclusion:**

DM2 influences PET-measured systolic function in patients without previous myocardial infarction independently from myocardial perfusion parameters. Our study supports the importance of DM2 as an independent risk factor for deteriorating systolic function.

**Electronic supplementary material:**

The online version of this article (doi:10.1007/s12350-016-0482-7) contains supplementary material, which is available to authorized users.

## Introduction

The importance of type 2 diabetes mellitus (DM2) in cardiovascular disease, ranging from endothelial dysfunction^1^ to heart failure, has been of great interest for clinicians. During the last 50 years, the cardiac disease profile has substantially shifted from a high acute mortality in the setting of myocardial infarction (MI) to a substantial decrease in first-event mortality and an increasing number of patients with progressive ventricular dysfunction and end-stage cardiac disease. Therefore, there is an interest in the independent influence of DM2 in coronary artery disease (CAD)-related outcomes. So far, the influence of DM2 on ventricular function is suggested to be mediated by the hampering of coronary vasculature dilatory function and microvascular involvement with further death risk modification dependent on its magnitude.^2^ However, a direct influence in the myocardium has also been proposed to underlie the diabetic cardiomyopathy phenotype.^3^–^7^ PET quantitative myocardial perfusion scanning constitutes a state-of-the-art technique for the evaluation of myocardial blood flow during rest (rMBF) and stress (sMBF) and for the determination of the myocardial perfusion reserve (MPR). Moreover, gated PET represents a validated technique for systolic function assessment through the left ventricle ejection fraction (LVEF).^8^ The present study aimed to evaluate whether there is a relationship between DM2 and systolic function when accounting for absolute quantitative perfusion (sMBF and MPR) and traditional cardiovascular risk factors, using Nitrogen-13 ammonia PET.

## Methods

### Population

We retrospectively analyzed a prospectively constructed database with data from 2012 to 2014. From the patients referred for PET myocardial perfusion scanning for evaluation of known or suspected CAD, we selected patients without a previous myocardial infarction as registered in the patients’ clinical history and confirmed with the absence of fixed perfusion defects in the scan results. In total, 585 patients were included for the analysis and demographic data regarding gender, age, body mass index, and cardiovascular risk factors including hypertension, hypercholesterolemia, smoking status, and DM2 were extracted from the electronic file system. DM2 was operationalized as a dichotomous variable for the presence or absence of the disease.

### PET Acquisition

Every patient underwent a two-phase (rest and adenosine stress) PET scan with the use of Nitrogen-13 ammonia as the perfusion radiotracer. All image data were acquired in list mode on a Siemens Biograph-16 TruePoint PET/CT (Siemens Healthcare, Knoxville, USA) with the TrueV option (the axial field of view of 21.6 cm). This 3D system consists of a 16-slice CT and a PET scanner with four rings of lutetium oxyorthosilicate (LSO) detectors. Patients were instructed to fast overnight and to avoid the consumption of methylxantine-, caffeine-containing beverages or medications for 24 hours before the study. Previous to the rest perfusion phase of the scans, a CT-based transmission scan (130 kVp; 25 ref.mAs; helical scan mode with a pitch of 0.95) was obtained during normal breathing for correction of photon attenuation for all PET acquisitions. The automatic co-registration of the CT attenuation map with the PET images was verified visually and alignment was corrected when necessary by an experienced nuclear medicine technician. During rest, myocardial perfusion was assessed using 300 MBq of Nitrogen-13 ammonia. Imaging lasted for 12 minutes and began simultaneously with peripheral injection of the radiotracer. The Nitrogen-13 ammonia was administered as a single intravenous bolus (8-10 s with infusion rate 0.4 mL/second) followed by a 10 mL saline flush. Pharmacologic stress imaging was performed one minute later and beginning with a 6-minutes adenosine infusion through a peripheral vein (140 µg/kg/minute). A second dose of 400 MBq Nitrogen-13 ammonia was injected in the fourth minute of the adenosine administration and image acquisition was started, in the same way, simultaneously with the radiotracer bolus. Static, dynamic, and 16-bin ECG-gated images were generated from the list mode data. Patient emission data was reconstructed using 3D attenuation-weighted ordered subsets expectation maximization (OSEM3D) reconstruction with 168 × 168 matrix, zoom 2, Gaussian filter with a full width at half maximum of 5 mm, 2 iterations, and 21 subsets for gated and dynamic images and TrueX (OSEM3D with PSF) reconstruction with 256 × 256 matrix, zoom 2, Gaussian filter of 4 mm, 4 iterations, and 8 subsets for static images. CT-based attenuation, scatter, decay, and random corrections were applied to the reconstructed images. Dynamic images were reconstructed with 25 frames for rest: 1 × 10, 12 × 5, 2 × 10, 7 × 30, 2 × 60, and 1 × 180 seconds, and 26 frames for stress: delay 90, 1 × 30, 1 × 10, 12 × 5, 2 × 10, 7 × 30, 2 × 60, and 1 × 180 seconds.

### Quantitative Perfusion

Based on the dynamic subsets, left ventricular contours were assigned automatically using the SyngoMBF software (Siemens Medical Solutions, Berlin, Germany) with minimum observer intervention when appropriate. With a previously described 2-compartment kinetic model for Nitrogen-13 ammonia, stress and rest flow values in mL/g/minute were computed for each sample on the polar map through the resulting time-activity curves for global quantification.^9^ Myocardial perfusion reserve (MPR) was calculated as the ratio between the MBF during stress (sMBF) and MBF during rest (rMBF) and therefore expressed adimensionally. rMBF was corrected for the rate pressure product (RPP) as previously described. The total MPR and sMBF were calculated within the whole left ventricular region as parameters of interest for our analysis.

### Left Ventricular Systolic Function

The list mode data were reconstructed in 16-bin ECG-gated images. The end-systolic and end-diastolic volumes were determined using Quantitative Gated SPECT 2012 (QGS) software (Cedars Sinai Medical Center). LVEF was expressed as the percentage of the end-diastolic volume ejected during systole.

### Statistical Analysis

Continuous variables were explored for sampling distribution through histograms and described as mean ± standard deviation. Dichotomous variables are described as the number of occurrences with their associated valid percentage graphically. The univariate analysis was performed through biserial correlations considering clinical variables, and a difference in means was explored through an independent samples *t* test. Further, a multiple linear regression analysis was performed by entering gender, age, hypercholesterolemia, hypertension, smoking habit, DM2, sMBF, and MPR as predictor (independent) variables and LVEF as outcome (dependent) variable. The model was bootstrapped based on 1000 samples with bias-corrected and accelerated 95% confidence intervals (95% BCa CIs) for robustness of B coefficients and significance estimators. A second step for the model was performed for testing a possible interaction between DM2 and both sMBF and MPR. *P* values less than .05 were considered significant. All statistical analyses were performed using SPSS (Released 2013. IBM SPSS Statistics for Windows, Version 22.0. Armonk, NY: IBM Corp.).

## Results

### Patient Characteristics

A total of 585 consecutive patients, 290 male and 295 female, with a mean age of 65.3 ± 9.9 and 67.4 ± 10.0 years were included in our analysis, respectively. There was a high prevalence of arterial hypertension while the rate of patients who were smokers or had diagnosed DM2 was comparable. Baseline demographic and perfusion variables are depicted in Table 1.

**Table 1:** Baseline population characteristics

Variable	Diabetics (n = 83)	Non-diabetics (n = 459)	*P* value
Men/women (n)	34/49	228/230	.153
Arterial hypertension	74%	55%	.010
Smokers	17%	16%	.870
Dyslipidemia	42%	37%	.325
Family history of CVD	31%	34%	.617
Rest MBF (mL/g/minute)	0.98 ± 0.22	1.00 ± 0.26	.244
Stress MBF (mL/g/minute)	2.01 ± 0.51	2.15 ± 0.54	.072
MPR	2.13 ± 0.48	2.21 ± 0.60	.172

*CVD*, cardiovascular disease; *MBF*, myocardial blood flow; *MPR*, myocardial perfusion reserve

### Quantitative Perfusion Assessment and Systolic Function

**Figure 1:**
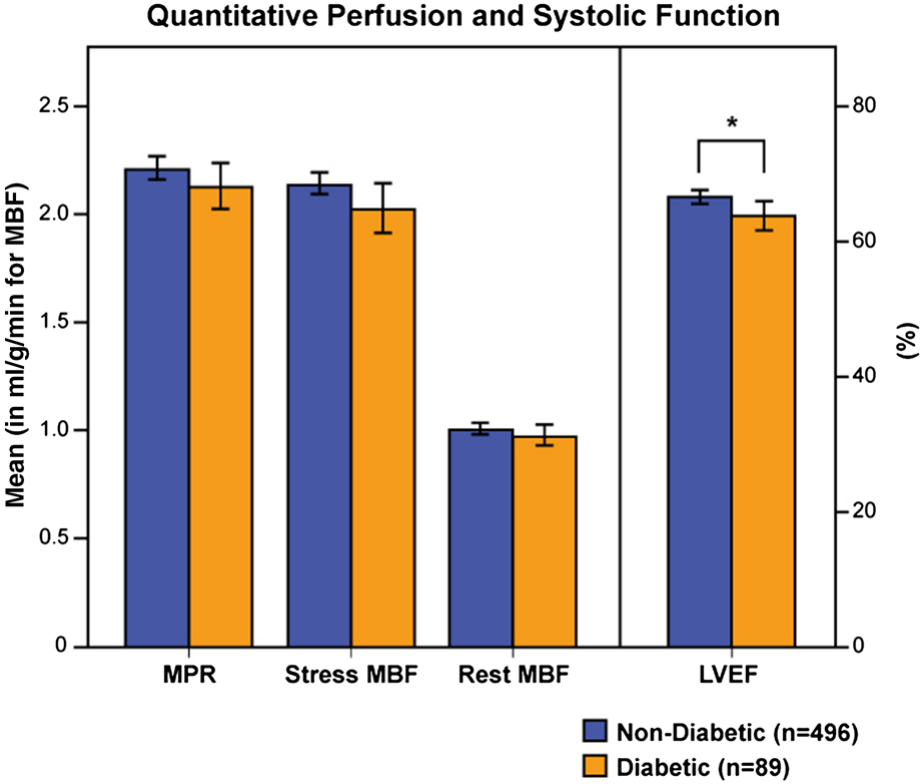
Quantitative perfusion and systolic function. The bar chart shows the mean value for quantitative perfusion results (*MPR*, myocardial perfusion reserve; *MBF*, myocardial blood flow) and systolic function (*LVEF*, left ventricle ejection fraction) in patients with (*orange*) and without DM2 (*blue*). The error bars represent 95% CIs

The population had a mean global rMBF of 1.00 ± 0.25 mL/g/minute and a sMBF of 2.11 ± 0.54 mL/g/minute. Consequently, the calculated ratio expressing global MPR was 2.18 ± 0.57. Mean LVEF was 65.7 ± 12.2% (Figure 1). The univariate analysis documented a significant biserial correlation between LVEF and DM2 (*r*_b_ = −0.14, *P* = .03) but not for smoking (*r*_b_ = −0.11, *P* = ns), dyslipidemia (*r*_b_ = −0.08, *P* = ns), or hypertension (*r*_b_ = 0.005, *P* = ns). The bootstrapped correlation between sMBF and LVEF was found to be significant (*r* = 0.330 [0.249, 0.420], *P* < .001). We evaluated the difference in mean LVEF between the patients with and without DM2 finding a statistically significant lower LVEF in the DM2 group *t* = −2.104 SE 1.269, *P* = .037, *d* = 0.25 (also depicted in Figure 1).

### Multiple Linear Regression

The bootstrapped multiple linear regression analysis determined a statistically significant relationship for gender (*P* = .001), sMBF (*P* = .001), and the presence of DM2 (*P* = .034) with LVEF. The statistical values for age (*P* = .218), dyslipidemia (*P* = .086), hypertension (*P* = .820), and smoking (*P* = .152) were not significant. The overall model was assessed through the change in coefficient of determination, *R* ^2^ = 0.162, *P* < .001. The corresponding coefficients and BCa 95% CIs are shown in Table 2. In the second step of the model, there was no significant interaction between DM2 and MPR (*P* = .19) or between DM2 and sMBF (*P* = .51), and there was no significant improvement of the model according to the change *R* ^2^.

**Table 2:** Multiple linear regression analysis (enter method) description

Dependent variable	Independent variable	*B*	(BCa) 95% CI		*R*	*P* value
			Lower bound	Upper bound		
LVEF^§^	Intercept	47.857	39.418	56.920	0.402	.001
	Gender	4.380	2.363	6.360		.001*
	Age	0.060	−0.039	0.155		.218
	Dyslipidemia	1.640	−0.167	3.443		.086
	Smoking	−1.725	−4.199	.810		.152
	Arterial hypertension	−0.192	−2.070	1.701		.820
	DM2	−2.599	−5.125	−.119		.034*
	sMBF	5.830	3.505	9.549		.001*
	MPR	−0.003	−3.870	.210		.965
Second step (model 1 + interaction)						
	Interaction DM2 × sMBF	1.671	−3.774	7.630		.512

*BCa*, bias-corrected accelerated; *DM2*, type 2 diabetes mellitus; *LVEF*, left ventricular ejection fraction; *MPR*, myocardial perfusion reserve; *sMBF*, stress myocardial blood flow

^§^Bootstrap based on 1000 samples

* *P* < .05

## Discussion

We have documented significant relations between cardiovascular risk factors, quantitative perfusion variables, and left ventricular systolic function in a large sample of patients referred to non-invasive evaluation in a specialized imaging center for suspected CAD. Our results evidenced a statistically significant relationship between the presence of DM2 and a decrease in LVEF independent of absolute quantitative perfusion variables (MPR and sMBF) and additional relevant risk factors (smoking habit, hypertension, and dyslipidemia).

When considering our population of patients without a previous myocardial infarction, these results line up with the current view of diabetic cardiomyopathy,^6^ which conveys a possible direct mechanistic relation between the pathophysiological state of diabetic patients and their corresponding left ventricular systolic function.^7^

Diabetic cardiomyopathy is defined as “a distinct entity characterized by the presence of abnormal myocardial performance or structure in the absence of epicardial coronary artery disease, hypertension, and significant valvular disease.”^4^,^5^ A multitude of direct pathophysiological mechanisms, such as direct effects of hyperglycemia, lipotoxicity, advanced glycation endproducts (AGEs) deposition, microvascular refraction, insulin resistance, and hyperinsulinemia have been proposed to contribute to the condition, although the exact pathogenesis is still to be elucidated.^7^ Diabetic cardiomyopathy has been proposed to arise from the additive effect of processes like myocardial interstitial deposition of AGEs, cardiomyocyte apoptosis, and reactive interstitial fibrosis with a deleterious effect that, in parallel with the (micro)vascular affection, disturb systolic, and diastolic function.^6^ Therefore, the main described mechanism of deterioration of the left ventricular function may not be the hampering of coronary vasculature dilatory function (linked to ischemic findings and/or microvascular dysfunction) alone.^2^

Previous research has reported a difference in systolic function measured through LVEF between patients with and without DM2 in the of setting patients without CAD.^6^ Although we found a similar difference in LVEF between the two groups, the effect size calculation showed that, in the univariate analysis, this corresponds to a rather small effect. As such, we incorporated the variable into a statistical model to generate a comprehensive overview of its importance in relation to systolic function (evaluated through LVEF) in relation to other important factors including: quantitative perfusion parameters sMBF and MPR, both of which consider hampered myocardial perfusion at all levels of the coronary vasculature ranging from epicardial significant or non-significant stenoses down to microvascular or endothelial dysfunction, and other conventional cardiovascular risk factors (age, gender, smoking status, hypercholesterolemia, arterial hypertension, or significant family history for CAD).

Interestingly, although there was a trend towards significance for the presence of dyslipidemia in the analysis, none of the otherwise generally considered cardiovascular risk factors yielded significance in our study. We consider that this supports the notion that the effect of DM2 may be of greater importance regarding systolic function assessment. A complementary explanation may be that other risk factors, such as age, dyslipidemia, arterial hypertension, and smoking probably hamper systolic function through their pathological effect on coronary vasodilatory function (endothelial dysfunction leading to coronary [micro]vascular “stiffness”), which again was accounted for in our analysis through the included perfusion variables (sMBF and MPR). The lack of significance for the interaction term (DM2 and sMBF) suggested that the significant effect found for DM2 was not explained by mediation through myocardial perfusion. Other effects such as cardiac sympathetic innervation dysfunction^10^ may play an etiologic role and further research in this area is warranted.

As evidenced by the multiple regression analysis, DM2 showed a relative greater influence over LVEF than MPR, but more discrete than gender or sMBF according to the adjusted B coefficients, as shown in Table 2. This may suggest that DM2 treatment should constitute a primordial target for preservation of LVEF, as much as perfusion optimization.

Recent evidence has demonstrated that the rate of cardiac death in diabetic patients without known CAD was low in the presence of a preserved MPR^2^ and that this might partially explain the inconsistent relationship between diabetes mellitus and cardiac risk. We consider that our explored independent relationship between DM2 and systolic function may also play a role in it.

The variable gender yielded significance in this analysis. This was expected when considering previous evidence of male-female differences in LVEF due to intrinsic anatomic and physiologic differences linked to the calculation of ejection fraction.^11^

Previous studies have repeatedly proven the relationship between PET-derived perfusion and ventricular function.^12^–^14^ Our study additionally showed a relative greater importance of sMBF (*B* = 7.133 [4.550, 9.604], *P* = .001) when compared to MPR (by inclusion in the same regression model). This finding is in line with recent reports proposing sMBF (hyperemic MBF) as a better measure of perfusion in patients with suspected CAD.^15^–^17^

Our results add to the body of evidence suggesting a decrease in systolic function in relation to the presence of DM2. Additionally, they describe proof of its statistically assessed independent influence when compared to other risk factors.

## New Knowledge Gained

This study demonstrated a relation between type 2 diabetes mellitus and systolic function in patients without previous myocardial infarction independently from and not mediated by quantitative perfusion results. Additionally, to the previously reported relationship between PET-derived perfusion and ventricular function, the present study showed a relative greater influence of stress myocardial blood flow compared to the myocardial perfusion reserve on systolic function.

## Limitations

Our results are limited by the availability of alternative parameters to evaluate the status of the diabetic patients such as HbA1c or time from diagnosis. Also, it would have been of benefit to consider a complementary technique for the evaluation of ventricular function. Nevertheless, the technique described constitutes a valid one for the evaluation of systolic function and the reference standard for perfusion assessment in absolute terms. Another limitation would be that we did not include data related to invasive angiographic evaluation since only a small proportion of the sample underwent the procedure, even so, univariate correlations between quantitative perfusion and systolic function were similar in these patients and ischemic burden may have overshadowed other potential effects. Additionally, regional flow was not considered separately and although of interest, we aimed to account for the global perfusion status and its influence on the ventricular function. Therefore, the influence of specific regional measures cannot be evaluated from this study alone. Finally, our population showed a discrete prevalence of DM2 patients, which might not concur with DM2 prevalence in patients referred to cardiac PET imaging; nevertheless, it portraits the prevalence in the general population as well as in previous published reports.^13^,^14^,^18^

## Conclusion

Diabetes mellitus significantly influences PET-measured systolic function in patients without a previous myocardial infarction, independently from myocardial perfusion parameters. Our study supports the importance of diabetes mellitus as an independent risk factor for deteriorating systolic function.

## Electronic supplementary material

Below is the link to the electronic supplementary material.

Supplementary material 1 (PPTX 274 kb)

## Abbreviations

DM2: Type 2 diabetes mellitus
PET: Positron emission tomography
PET/CT: Positron emission tomography/computed tomography
CAD: Coronary artery disease
LVEF: Left ventricular ejection fraction
MI: Myocardial infarction
sMBF: Stress myocardial blood flow
rMBF: Rest myocardial blood flow
MPR: Myocardial perfusion reserve
RPP: Rate pressure product
