# Supplementary material for: Type 2 diabetes mellitus correlates with systolic function during myocardial stress perfusion scanning with Nitrogen-13 ammonia PET
Source: J Nucl Cardiol. 2016 Apr 15;24(4):1305–11. doi: 10.1007/s12350-016-0482-7 (PMC5548822; doi:10.1007/s12350-016-0482-7)
Supplement: Supplementary file 1 — Supplementary material 1 (PPTX 274 kb) [file 12350_2016_482_MOESM1_ESM.pptx]

## Slide 1
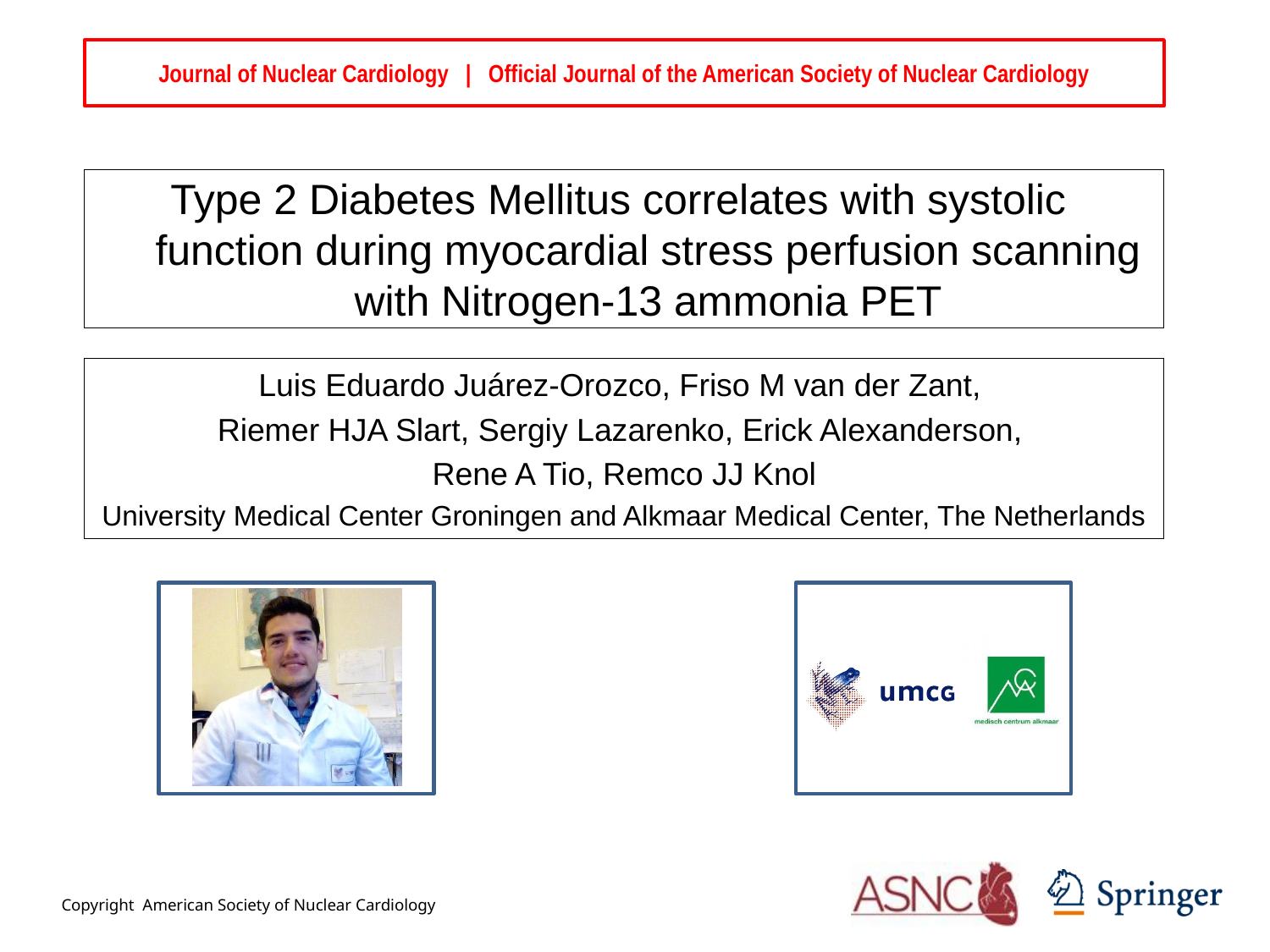

Journal of Nuclear Cardiology | Official Journal of the American Society of Nuclear Cardiology
# Type 2 Diabetes Mellitus correlates with systolic function during myocardial stress perfusion scanning with Nitrogen-13 ammonia PET
Luis Eduardo Juárez-Orozco, Friso M van der Zant,
Riemer HJA Slart, Sergiy Lazarenko, Erick Alexanderson,
Rene A Tio, Remco JJ Knol
University Medical Center Groningen and Alkmaar Medical Center, The Netherlands
Copyright American Society of Nuclear Cardiology

## Slide 2
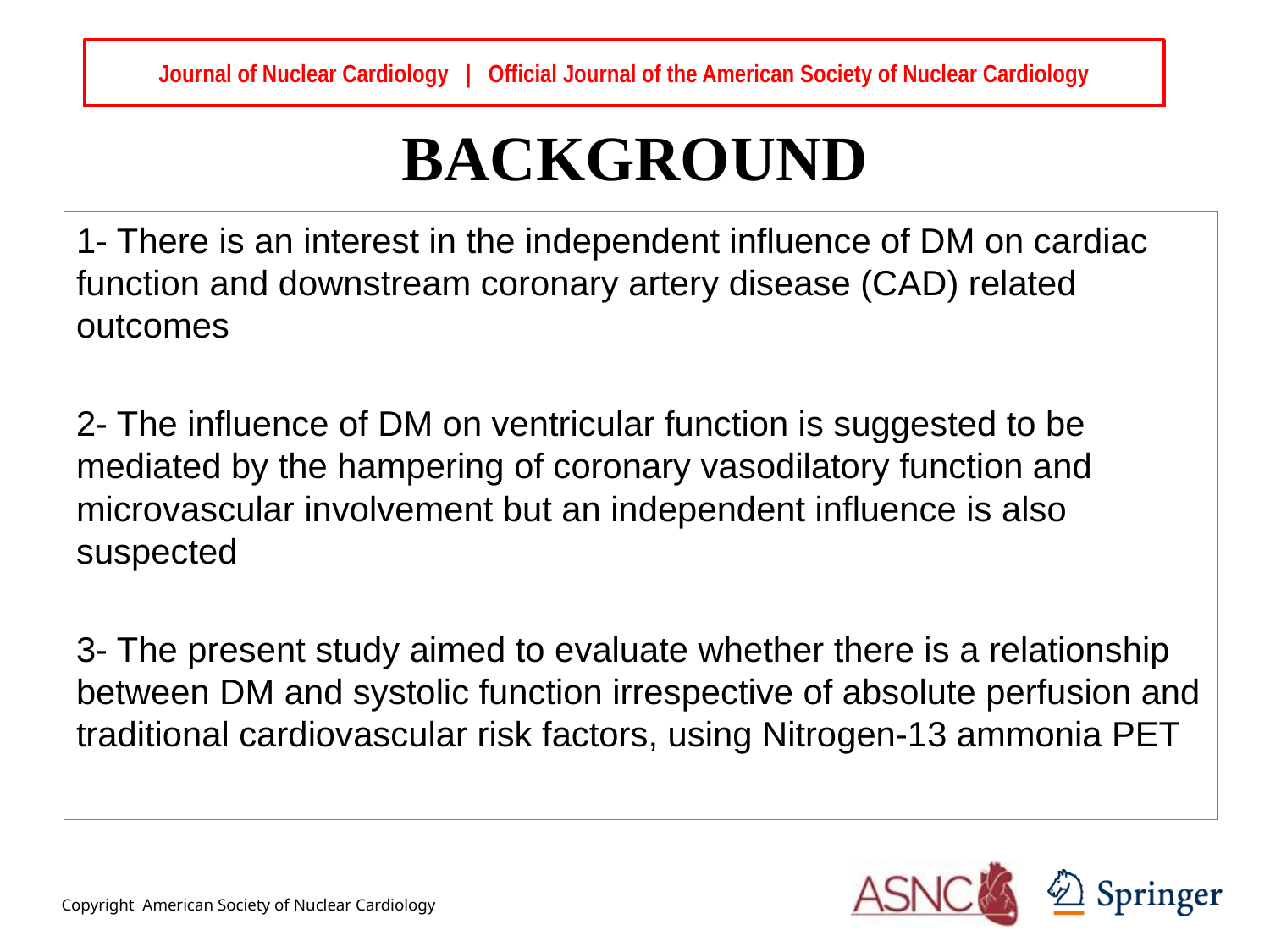

Journal of Nuclear Cardiology | Official Journal of the American Society of Nuclear Cardiology
# BACKGROUND
1- There is an interest in the independent influence of DM on cardiac function and downstream coronary artery disease (CAD) related outcomes
2- The influence of DM on ventricular function is suggested to be mediated by the hampering of coronary vasodilatory function and microvascular involvement but an independent influence is also suspected
3- The present study aimed to evaluate whether there is a relationship between DM and systolic function irrespective of absolute perfusion and traditional cardiovascular risk factors, using Nitrogen-13 ammonia PET
Copyright American Society of Nuclear Cardiology

## Slide 3
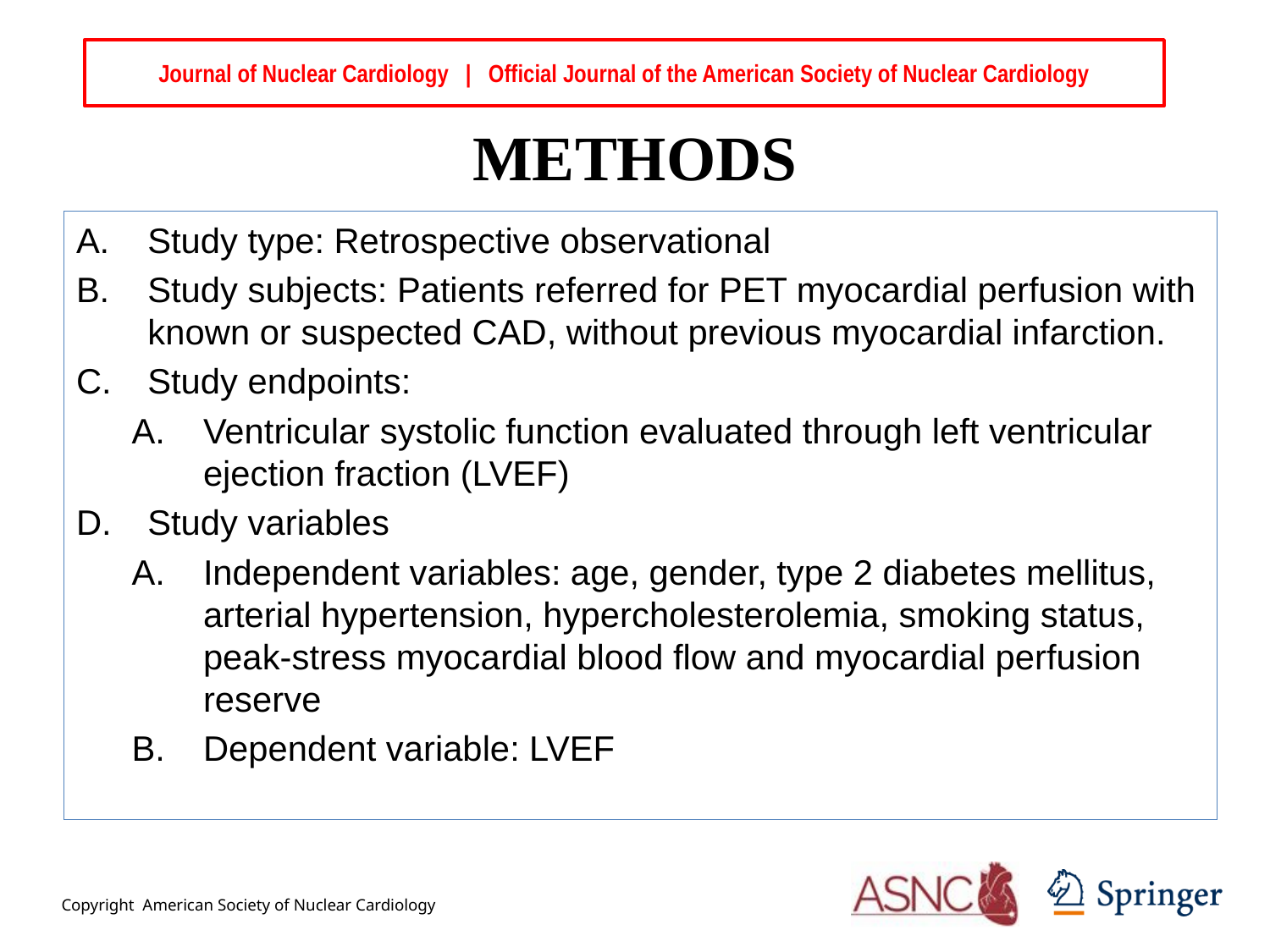

Journal of Nuclear Cardiology | Official Journal of the American Society of Nuclear Cardiology
# METHODS
Study type: Retrospective observational
Study subjects: Patients referred for PET myocardial perfusion with known or suspected CAD, without previous myocardial infarction.
Study endpoints:
Ventricular systolic function evaluated through left ventricular ejection fraction (LVEF)
Study variables
Independent variables: age, gender, type 2 diabetes mellitus, arterial hypertension, hypercholesterolemia, smoking status, peak-stress myocardial blood flow and myocardial perfusion reserve
Dependent variable: LVEF
Copyright American Society of Nuclear Cardiology

## Slide 4
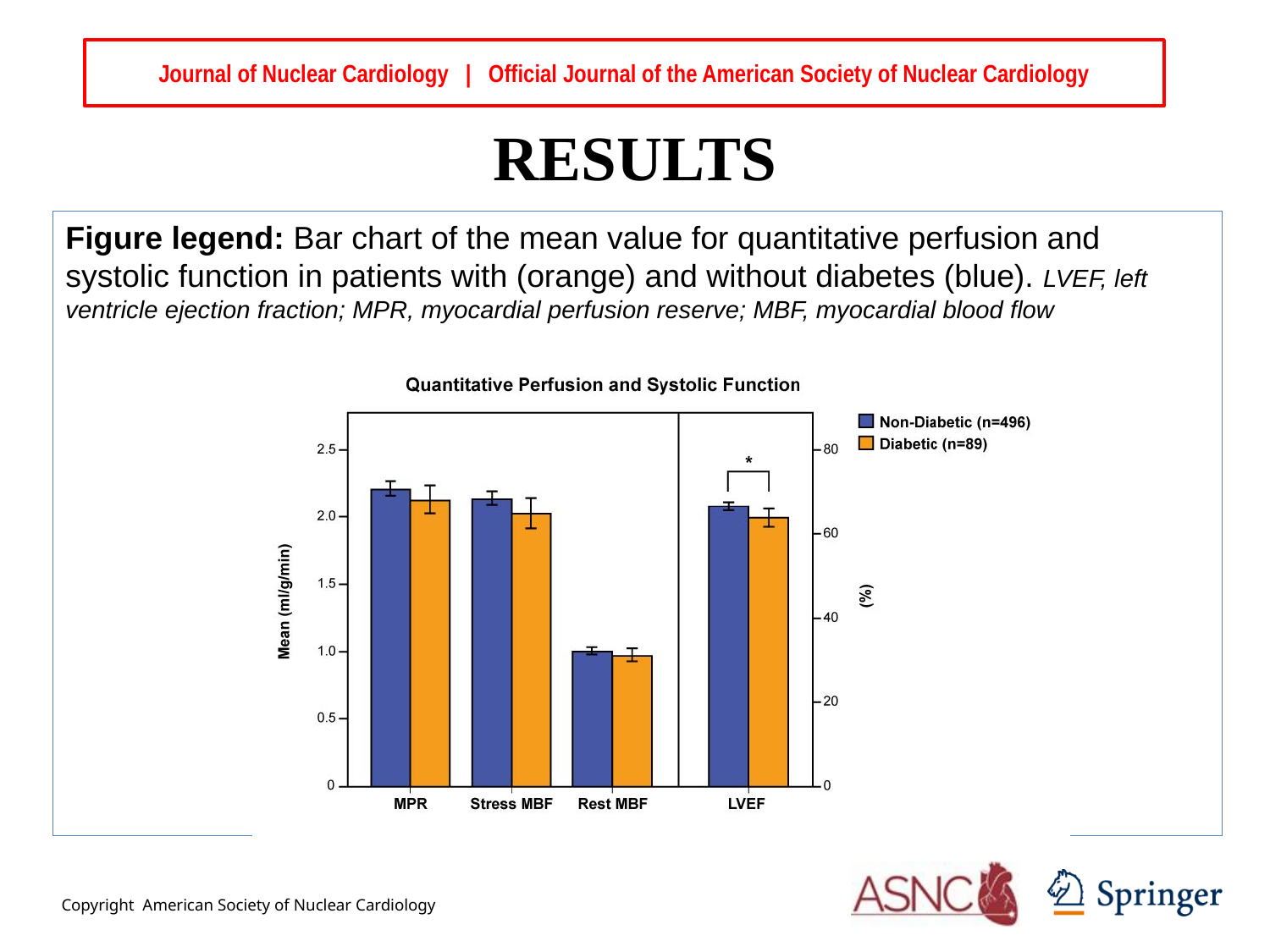

Journal of Nuclear Cardiology | Official Journal of the American Society of Nuclear Cardiology
# RESULTS
Figure legend: Bar chart of the mean value for quantitative perfusion and systolic function in patients with (orange) and without diabetes (blue). LVEF, left ventricle ejection fraction; MPR, myocardial perfusion reserve; MBF, myocardial blood flow
Copyright American Society of Nuclear Cardiology

## Slide 5
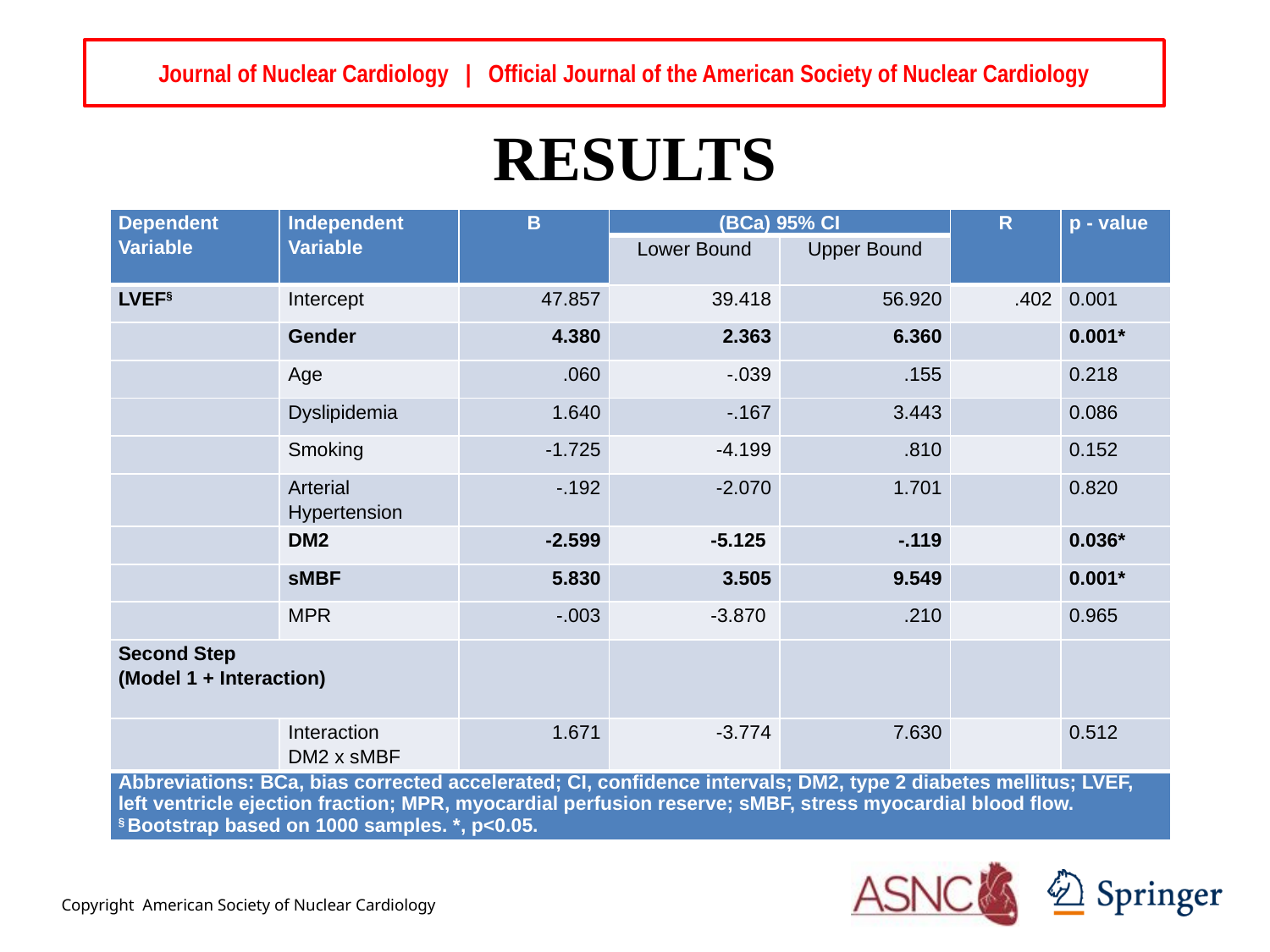

Journal of Nuclear Cardiology | Official Journal of the American Society of Nuclear Cardiology
# RESULTS
| Dependent Variable | Independent Variable | B | (BCa) 95% CI | | R | p - value |
| --- | --- | --- | --- | --- | --- | --- |
| | | | Lower Bound | Upper Bound | | |
| LVEF§ | Intercept | 47.857 | 39.418 | 56.920 | .402 | 0.001 |
| | Gender | 4.380 | 2.363 | 6.360 | | 0.001\* |
| | Age | .060 | -.039 | .155 | | 0.218 |
| | Dyslipidemia | 1.640 | -.167 | 3.443 | | 0.086 |
| | Smoking | -1.725 | -4.199 | .810 | | 0.152 |
| | Arterial Hypertension | -.192 | -2.070 | 1.701 | | 0.820 |
| | DM2 | -2.599 | -5.125 | -.119 | | 0.036\* |
| | sMBF | 5.830 | 3.505 | 9.549 | | 0.001\* |
| | MPR | -.003 | -3.870 | .210 | | 0.965 |
| Second Step (Model 1 + Interaction) | | | | | | |
| | Interaction DM2 x sMBF | 1.671 | -3.774 | 7.630 | | 0.512 |
| Abbreviations: BCa, bias corrected accelerated; CI, confidence intervals; DM2, type 2 diabetes mellitus; LVEF, left ventricle ejection fraction; MPR, myocardial perfusion reserve; sMBF, stress myocardial blood flow. § Bootstrap based on 1000 samples. \*, p<0.05. | | | | | | |
Copyright American Society of Nuclear Cardiology

## Slide 6
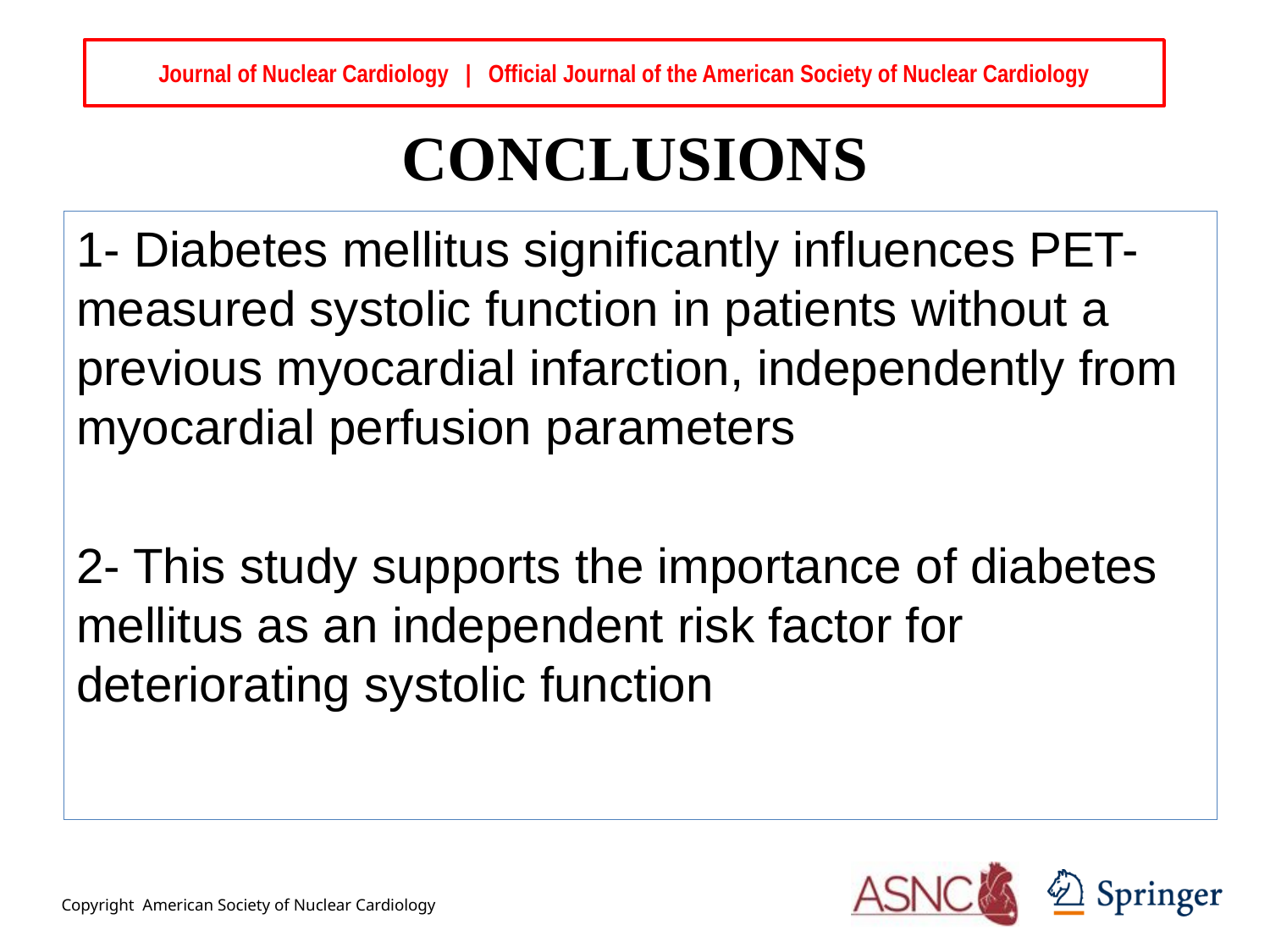

Journal of Nuclear Cardiology | Official Journal of the American Society of Nuclear Cardiology
# CONCLUSIONS
1- Diabetes mellitus significantly influences PET-measured systolic function in patients without a previous myocardial infarction, independently from myocardial perfusion parameters
2- This study supports the importance of diabetes mellitus as an independent risk factor for deteriorating systolic function
Copyright American Society of Nuclear Cardiology
